# Reliability of the Footscan® Platform System in Healthy Subjects: A Comparison of without Top-Layer and with Top-Layer Protocols

**DOI:** 10.1155/2017/2708712

**Published:** 2017-09-11

**Authors:** Chao Xu, Xin-Xin Wen, Lu-Yu Huang, Lei Shang, Zhao Yang, Ya-Bo Yan, Wei Lei

**Affiliations:** ^1^Department of Orthopedics, Xijing Hospital, Fourth Military Medical University, Xi'an 710032, China; ^2^Department of Orthopedics, No. 463 Hospital of Chinese PLA, Shenyang 110042, China; ^3^Department of Health Statistics, Faculty of Preventive Medicine, Fourth Military Medical University, Xi'an 710032, China

## Abstract

The Footscan platform is a useful tool for plantar pressure measurement. However, there is still controversy over whether or not the platform should be covered by top-layer during the test. This study was designed to compare the reliability of the Footscan platform and identify the differences of the foot loading parameters between without top-layer (WOT) and with top-layer (WT) protocols. Measurements were taken from thirty-two healthy subjects. Participants were tested with a Footscan platform using the WOT and WT protocols. Three trials were performed during two separate testing sessions with a 7-day interval. Peak pressure, contact time, contact area, and pressure-time integral at ten foot zones were recorded and calculated for intra- and intersession reliability using intraclass correlation coefficients (ICCs) and coefficients of variation (CVs). The reliability and values of the analyzed parameters for the two protocols were compared. Both protocols produced a moderate to good level of intra- and intersession reliability. Compared with the WT protocol, the WOT protocol showed higher ICCs, lower CVs, and higher values in most of the parameters analyzed. The results suggest that the WOT protocol showed better reliability than the WT protocol. We recommend not using the top-layer when performing the plantar pressure test.

## 1. Introduction

With the development of microcomputer technology, plantar pressure measurement systems are being used more frequently in research and clinical practice. The systems can be used to distinguish between normal and pathological gait [1], design foot orthoses [2], predict risk factors for lower extremity injuries [3], assess progress of disorders [4], evaluate the effect of treatment [5], and so forth. For these measurements to have clinical application, it is necessary to ensure that the systems can achieve a high level of reliability, accuracy, and consistency for plantar pressure measurement on different occasions [6].

At present, the commercially available pressure measuring systems include in-shoe measurement systems (Novel Pedar®, TekScan F-Scan®, RSscan Insole®, WalkinSense®, and IBV Biofoot®) and platform systems (Novel Emed®, TekScan MatScan®, and RSscan Footscan) [7]. Most of them have been validated as reliable tools for quantifying dynamic plantar pressure [7–16]. Footscan platform is one of the most commonly used pressure measuring systems. However, there is still controversy over whether or not the Footscan platform should be covered by top-layer during the test.

After a comprehensive search, we found that 36 investigations using the Footscan platform systems have been published in the PubMed database by the end of March, 2017. 25% (9 of 36) of these studies covered the platform with a thin top-layer made from ethylene-vinyl acetate copolymer (EVA) or other materials. These researchers believed the disguised platform can prevent the subjects from adjusting their normal walking patterns induced by the visual targeting of the pressure plate [17, 18]. However, 75% (27 of 36) of these studies did not use any top-layer. Some researchers have reported that visual targeting during walking did not affect the magnitude or variability of the ground reaction force when the study design was tailored to the subjects' gait variables [19–21]. In addition, a factor that may increase the number of rejected trials in plantar pressure test is the prerequisite that the subject's foot must land completely within the bounds of the pressure plate during stance. A pressure plate without top-layer can help the subject's foot be entirely on a pressure plate during test and reduce the potential trials' number, which means being less time-consuming and less strenuous and is important for the pathological populations [19, 20].

Our research team have assessed the reliability of the Footscan platform system and identified the range of loading parameters observed in the normal foot without using any top-layer [16]. However, from a literature survey, it appears that none of the previous investigators is concerned with the effects of top-layer on reliability of the Footscan platform system during barefoot walking. This lack of information becomes a barrier for using the top-layer in the measurement of plantar pressure. To use the top-layer or not, that is a question. Therefore, the primary aim of this study was to compare the reliability of the Footscan platform system between the WT and WOT protocols. The second objective was to detail differences of the foot loading parameters between the two protocols.

## 2. Materials and Methods

### 2.1. Subjects

Thirty-two healthy volunteers (*n* = 32) were recruited for assessment from the local area. Participants included in the present study were healthy and capable of ambulating independently and aged between 18 and 40 years. Participants were excluded if they suffered from foot pain and/or injuries within the previous 6 months, had any previous surgeries to the foot and ankle, limb length discrepancies, or foot deformities, or had any clinical problems that could potentially affect their gait. Gender, age (years), body mass (kg), height (cm), and body mass index (BMI) (kg/m^2^) were recorded for each subject at baseline. The study was approved by the Ethical Committee of the Fourth Military Medical University. All experiments were performed in accordance with relevant guidelines and regulations. Written informed consent was obtained from each subject prior to testing.

### 2.2. Experimental Apparatus and Set-Up

Dynamic plantar pressure parameters were recorded using a Footscan pressure plate (RSscan International, Olen, Belgium, 2096 mm × 472 mm × 18 mm, with 16384 resistive sensors arranged in a 256 × 64 matrix at a resolution of 2 sensors/cm^2^, data acquisition frequency: 125 Hz, pressure range: 0–200 N/cm^2^), which was connected to a computer using the supplied cable. The platform was located at the center of a carpet with the same external dimension to provide a “complete platform” 4 m in length [22]. According to the manufacturer's manual, the Footscan system was calibrated before each measurement session. During calibration, the subject's weight was entered into the computer and then the subject was asked to walk across the plate at preferred speed while barefoot. After that, the analysis software will determine a recalibration factor which is used to calibrate future measurements.

### 2.3. Procedure

**Figure 1:**
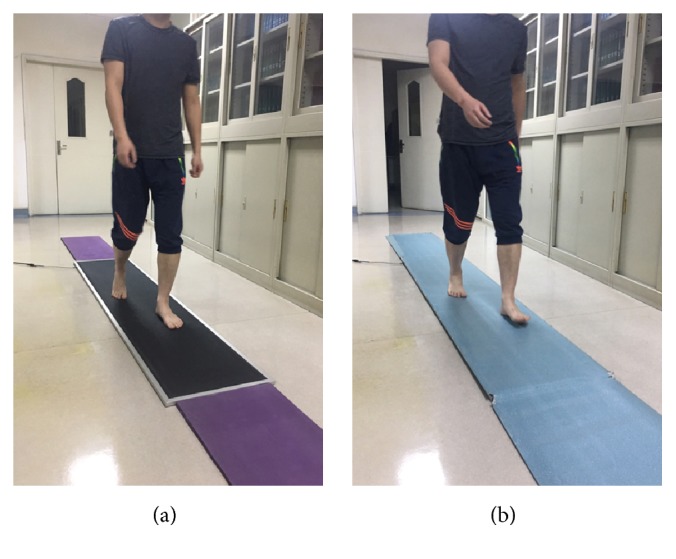
Illustration of the experimental protocols. (a) The without top-layer protocol; (b) the with top-layer protocol.

Testing sessions were conducted on two occasions 7 days apart. In each session, participants were tested with two protocols. For the WT protocol, the platform was covered with a top-layer made from EVA material (hardness: Shore A 70). For the WOT protocol, the platform was covered with nothing (Figure 1). The sequence of protocols was randomly distributed over the patients. In each protocol, three representative and reliable trials were recorded for each participant [6, 12]. A representative trial should meet the following criteria: (1) at least two complete footprints, (2) a heel-strike pattern, and (3) no obvious adjustment in gait pattern to contact the plate [23]. A trial was repeated if the researchers observed an atypical foot placement on the platform. All the subjects received clear instructions about the testing protocols. Meanwhile, they were asked to wear casual loose fitting clothing that did not impede lower limb motion.

Before data collection, all the participants initially completed 10-minute acclimatization walking trails along the measuring platform. Based on individual stride and step length obtained during acclimatization trials, each participant determined a suitable starting position to ensure that 3 steps were taken prior to platform contact [21]. This approach ensured that data were collected during mid-gait which can minimize the effect of acceleration and deceleration at the start and end of each walk [24]. Then, the subjects were asked to perform pedobarographic tests barefoot at their comfortable walking pace. One step with each foot was recorded per walking trial, and three steps with each foot were recorded per session. To prevent fatigue, each participant was asked to take a rest of 3 minutes between each trial [22]. Trial order was randomized between participants.

### 2.4. Data Processing

**Figure 2:**
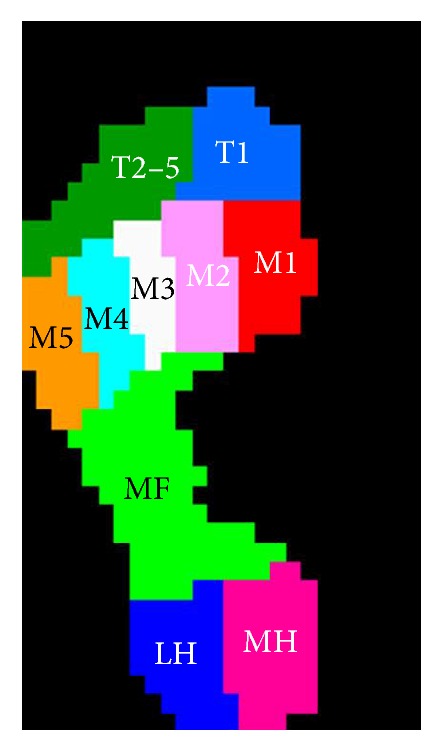
Schematic diagram for the 10 subdivided zones of the foot applied in the current study. The subdivided zones were (T1) hallux, (T2–5) toes 2–5, (M1) first metatarsal, (M2) second metatarsal, (M3) third metatarsal, (M4) fourth metatarsal, (M5) fifth metatarsal, (MF) midfoot, (MH) medial heel, and (LH) lateral heel.

The data were analyzed using Scientific Footscan Software (RSscan International). The software automatically divided the foot into 10 masked zones: hallux (T1), toes 2–5 (T2–5), first to fifth metatarsals (M1, M2, M3, M4, and M5), midfoot (MF), medial heel (MH), and lateral heel (LH) (Figure 2). Four of the clinically most relevant parameters were selected for evaluation: peak pressure (PP, kPa), contact time (CT, stance time%), contact area (CA, cm^2^), and pressure-time integral (PTI, kPa s). In total, 40 parameters were assessed: 4 foot loading variables, under 10 masked zones.

### 2.5. Statistical Analysis

Statistical analyses were performed using SPSS software (SPSS 19.0; SPSS Inc, Chicago, IL). The mean and standard deviation (SD) were calculated for each parameter and the data were examined for normality to check that they met the parametric assumptions.

To maintain independence of data only the left foot of each participant was chosen to be assessed [7, 25–27]. Intrasession reliability was evaluated using the intraclass correlation coefficients (ICCs) and coefficients of variation (CVs) across the three repeated trials within the same session. Intersession reliability was assessed using the average of the three trials in each session to calculate the ICCs and CVs. We considered ICC < 0.50 as poor, 0.50–0.75 as moderate, and >0.75 as good [7]. The type of ICC used for this analysis was a one-way random ICC, since the differences in results between testing sessions were random [28].

Then, to assess for systematic differences between sessions, for both protocols, paired *t*-tests were used to compare mean values of the foot loading parameters of interest for each masked zone. The maximum probability level to denote statistical significance was 0.05.

In addition, to detail the differences between the two protocols, paired *t*-tests were used to compare mean values (all six repeated trails on two sessions) of the foot loading parameters of interest for each masked zone. Furthermore, the differences between the WT and WOT protocols were verified by the (i) absolute (WOT − WT) and (ii) percentage [(WOT − WT) × 100/WOT] difference analyses [12]. Negative values indicate that the values in WT protocol were higher than those in the WOT protocol, while positive values indicate that the WOT protocol showed values higher than the WT protocol.

## 3. Results

### 3.1. Participants Characteristics

The mean (SD, range) age, body mass, height, and BMI of the participants were 26.4 (5.0, range 19 to 36) years, 69.6 (11.3, range 49.5 to 100.0) kg, 174.1 (6.9, range 159 to 185) cm, and 22.9 (3.1, range 18.7 to 31.6) kg/m^2^, respectively. Of the 32 subjects, 15 (46.9%) were female and 17 (53.1%) were male.

### 3.2. Intrasession Reliability

For the WOT protocol, the average ICCs and CVs values for all regions of the foot were 0.806 and 17.1%, respectively, for PP, 0.784 and 7.8% for CT, 0.890 and 6.7% for CA, and 0.760 and 17.7% for PTI. The regional intrasession ICCs for the PP were moderate in one (MF) and good in nine out of the ten masked zones. For the CT, the intrasession ICCs were moderate in two zones (T1 and T2–5) and good in the remaining eight zones. For the CA, all the regional intrasession ICCs were good. For the PTI, the intrasession ICCs were moderate in four zones (T1, T2–5, M1, and MF) and good in the remaining six zones (Table 1).

For the WT protocol, the average ICCs and CVs values for all regions of the foot were 0.684 and 20.7%, respectively, for PP, 0.734 and 9.0% for CT, 0.775 and 9.7% for CA, and 0.724 and 22.3% for PTI. The regional intrasession ICCs for the PP were good in one (T1) and moderate in nine out of the ten masked zones. For the CT, the intrasession ICCs were good in four zones (M1, M5, MH, and LH) and moderate in the remaining six zones. For the CA, the intrasession ICCs were good in five zones (T1, M1, MF, MH, and LH) and moderate in the remaining five zones. For the PTI, the intrasession ICCs were good in four zones (M3, M5, MH, and LH) and moderate in the remaining six zones (Table 1).

### 3.3. Intersession Reliability

For the WOT protocol, the average ICCs and CVs values for all regions of the foot were 0.843 and 11.5%, respectively for PP, 0.867 and 4.5% for CT, 0.889 and 4.5% for CA, and 0.813 and 14.2% for PTI. All the regional intersession ICCs for the PP, CT, CA, and PTI were good in the ten masked zones (Table 2).

For the WT protocol, the average ICCs and CVs values for all regions of the foot were 0.837 and 11.4%, respectively, for PP, 0.815 and 5.9% for CT, 0.828 and 5.3% for CA, and 0.807 and 16.6% for PTI. All the regional intersession ICCs for the PP and PTI were good. Meanwhile, the intersession ICCs for the CT were moderate in two (T2–5 and M3) and good in the remaining eight masked zones. For the CA, the intersession ICCs were moderate in two zones (M4 and M5) and good in the remaining eight zones (Table 2).

### 3.4. Systematic Differences in the Mean Values between Sessions

For both protocols, there were no systematic differences in mean values of the PP, CT, CA, and PTI between sessions (Tables 3 and 4).

### 3.5. Differences in the Values between the WOT and WT Protocols

The WOT protocol showed higher PP in all ten masked zones compared with the WT protocol, and the difference reached statistical significance in the T1, M2, M3, M4, MF, MH, and LH zones. The absolute differences of PP ranged from 2.4 (M5) to 36.0 kPa (M2). The percentage differences of PP ranged from 2.1% (M5) to 14.2% (T1) and the average percentage difference of PP between the two protocols was 9.4% (Table 5).

The WOT protocol showed higher CT in all ten masked zones compared with the WT protocol, and the difference reached statistical significance in the T1, T2–5, and M1 zones. The absolute differences of CT ranged from 0.2 (M5 and MH) to 6.5% (T2–5). The percentage differences of CT ranged from 0.3% (M5 and MH) to 15.6% (T2–5) and the average percentage difference of CT between the two protocols was 3.2% (Table 6).

Significantly higher CA in the T2–5, M1, M2, M3, and M4 zones were noted in the WOT protocol compared with corresponding values in the WT protocol. The absolute differences of CA ranged from −0.8 (MH) to 2.8 cm^2^ (T2–5). The percentage differences of CA ranged from −3.7% (MH) to 17.5% (T2–5) and the average percentage difference of CA between the two protocols was 4.8% (Table 7).

The WOT protocol showed higher PTI in all ten masked zones compared with the WT protocol, and the difference reached statistical significance in the T1, T2–5, M4, MH, and LH zones. The absolute differences of PTI ranged from 2.9 (T2–5) to 10.5 kPa s (M4). The percentage differences of CA ranged from 3.6% (M3) to 34.1% (T2–5) and the average percentage difference of CA between the two protocols was 13.9% (Table 8).

## 4. Discussion

Plantar pressure measurement is a useful evaluation tool for the patients with walking problems in research and clinical setting. As more and more clinical decisions and treatment strategies are made based on the data collected by the plantar pressure systems, the reliability and repeatability of the systems must be ascertained. Some researchers have reported that, for the same measuring system, different experimental protocols may affect its reliability and measurement values [6, 29]. The aim of this study was to compare the reliability of the Footscan platform system and detail the differences of the foot loading parameters of interest between the WOT and WT protocols. The results showed a generally moderate to good level of intra- and intersession reliability in both protocols, and the WOT protocol produced better reliability and higher values in most of the parameters of interest.

Regarding the intrasession reliability, the WOT protocol produced higher ICCs in 97.5% (39/40) and lower CVs in 82.5% (33/40) parameters of interest, comparing with the WT protocol. These results indicated that the WOT protocol produced better intrasession reliability than the WT protocol. According to the product manual, the Footscan platform system collects foot loading information using resistive pressure sensors. Therefore, for the cushioning effect of the EVA top-layer, we can speculate that the platform without top-layer will record higher plantar pressure parameters than the platform with top-layer under the same load. The results of this study confirmed our speculation. The WOT protocol showed higher values in 87.5% (35/40) parameters studied, comparing with the WT protocol, and the difference reached statistical significance in 50% (20/40) parameters. Some researchers [7, 9, 14, 30] have reported that areas with high loading characteristics showed a higher level of reliability than less loaded areas, which is in accordance with our findings. In the present study, we found that areas with higher PP, such as the M2, M3, MH, and LH zones, showed higher mean values of ICCs across variables of interest than the less loaded regions, such as the T2–5 and MF zones. The findings are clinically important because high foot loadings are good risk indicators for foot injuries [31, 32]. Therefore, a higher reliability in these regions is highly desirable for clinical applications [14].

In terms of intersession reliability, the WOT protocol produced higher ICCs in 62.5% (25/40) and lower CVs in 75% (30/40) parameters of interest, comparing with the WT protocol. These results showed that the WOT protocol had better intersession reliability than the WT protocol. It is worth noting that, for both protocols, the intersession ICCs are higher and CVs are lower than the corresponding intrasession ICCs and CVs in most of the parameters analyzed. According to Vallejo et al. [24], these differences may be because of minor unavoidable and expected physiological changes that occur during the walking process which can affect foot loading parameters. In light of that, a single trial is not enough, as physiological fluctuations between trials are not avoidable [24]. To achieve a high level of reliability, it is necessary to average over multiple trials.

Assessment for systematic differences between sessions indicated that, for both protocols, all the parameters of interest did not show any significant differences in mean values, which is consistent with the previous study [7]. The results suggest that a qualified platform such as Footscan system can achieve a satisfactory level of accuracy and reliability for plantar pressure measurement on different occasions.

**Figure 3:**
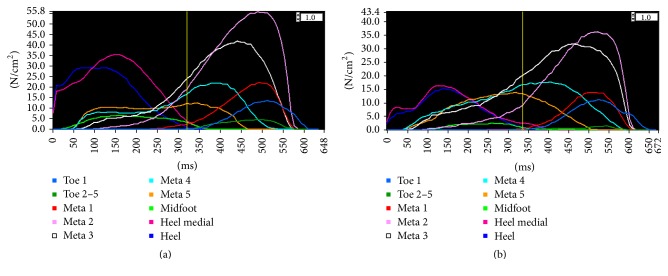
The curves of the peak pressure for the 10 masked zones of a representative subject in the present study. (a) The curves of the peak pressure in the without top-layer protocol; (b) the curves of the peak pressure in the with top-layer protocol. The subdivided zones were (Toe 1) hallux, (Toe 2–5) toes 2–5, (Meta 1) first metatarsal, (Meta 2) second metatarsal, (Meta 3) third metatarsal, (Meta 4) fourth metatarsal, (Meta 5) fifth metatarsal, (Midfoot) midfoot, (Heal medial) medial heel, and (Heel) lateral heel. 1 N/cm^2^ = 10 kPa.

The WOT and WT protocols showed similar pressure distribution and foot loading patterns. For both protocols, the higher PP values were recorded under the M2, M3, and MH regions, and the lower ones were found under the T2–5 and MF zones (Figure 3). These findings are in agreement with previous reports [8, 9, 11, 13, 26]. In addition, for both protocols, CT was longest in the metatarsal regions, and the metatarsal heads bore weight for 68.5% to 82.8% of the stance time in the WOT protocol and for 66.3% to 82.4% in the WT protocol, both of which are comparable with previous studies [8, 9, 11, 13]. In terms of CA, for both protocols, the MF, MH, and LH zones were the top 3 regions showing largest CA and the metatarsal regions had the smaller CA. Meanwhile, for both protocols, the PTI values were higher under the M2, M3, M4, and MH zones, and lower under the T2–5 and MF zones, which are also consistent with previous studies [8, 9, 11, 13]. The average percentage differences of PP, CT, CA, and PTI between the two protocols were 9.4%, 3.2%, 4.8%, and 13.9%, respectively. The results indicated that the top-layer had a greater impact on the values of the PTI and PP than that of the CA and CT. The differences between the two protocols suggest that the use of top-layer should be taken into consideration when comparing the data from studies with different testing protocols, especially for the data of PP and PTI.

There are some limitations of this study that need to be recognized. First, the subjects in this study did not have any problems with balance or gait, so our findings cannot necessarily be extrapolated to other clinical populations. Future researches should focus on the reliability of plantar pressure measurement in patients with gait problems. Second, different brands of systems may have different sensor technologies and performance characteristics, so the results of this study can only be considered when using the Footscan platform system. Finally, only one kind of top-layer was employed in this study, which may reduce the generalizability and the comparability of our findings.

## 5. Conclusions

In conclusion, the results of our study indicated that the WOT protocol had better reliability and higher values of foot loading parameters of interest than the WT protocol. We recommend not using the EVA top-layer when performing the plantar pressure test with the Footscan platform system. More research is required to determine the influences of other kinds of top-layers on the reliability and values of the foot loading parameters in patients with gait problems.

## Figures and Tables

**Table 1:** Regional intrasession ICCs and CVs for plantar loading measures in the WOT and WT protocols.

Variable	Zone	WOT		WT	
		ICCs	CVs	ICCs	CVs
PP	T1	0.835	19.8	0.831	23.4
	T2–5	0.756	22.0	0.520	37.2
	M1	0.768	19.8	0.746	21.2
	M2	0.914	13.5	0.688	17.4
	M3	0.859	15.0	0.701	18.8
	M4	0.803	14.0	0.667	17.6
	M5	0.783	16.1	0.609	20.3
	MF	0.715	25.2	0.613	23.7
	MH	0.816	12.4	0.735	13.4
	LH	0.813	13.1	0.725	13.7

CT	T1	0.721	16.3	0.715	15.3
	T2–5	0.651	19.7	0.622	26.5
	M1	0.818	10.3	0.800	12.5
	M2	0.794	2.9	0.682	7.9
	M3	0.783	2.4	0.699	2.9
	M4	0.789	2.6	0.687	3.5
	M5	0.775	3.7	0.768	3.5
	MF	0.813	7.7	0.720	7.7
	MH	0.834	5.9	0.821	5.3
	LH	0.858	6.0	0.824	5.1

CA	T1	0.851	7.8	0.775	10.1
	T2–5	0.846	10.4	0.683	24.5
	M1	0.888	9.1	0.804	12.1
	M2	0.858	6.2	0.701	8.1
	M3	0.863	6.5	0.712	7.4
	M4	0.873	5.7	0.695	7.4
	M5	0.884	6.8	0.739	8.8
	MF	0.949	7.4	0.893	9.9
	MH	0.947	3.5	0.816	4.8
	LH	0.940	3.8	0.932	3.5

PTI	T1	0.626	25.1	0.602	32.0
	T2–5	0.698	27.7	0.617	37.6
	M1	0.750	16.6	0.725	19.6
	M2	0.825	13.0	0.739	17.8
	M3	0.833	12.0	0.767	16.4
	M4	0.769	14.3	0.743	17.8
	M5	0.757	15.3	0.766	19.0
	MF	0.721	22.8	0.705	23.2
	MH	0.808	15.1	0.780	20.7
	LH	0.814	15.4	0.791	18.5

ICCs: intraclass correlation coefficients, CVs: coefficient of variations, WOT: without top-layer, WT: with top-layer, PP: peak pressure, CT: contact time, CA: contact area, PTI: pressure-time integral, T1: hallux, T2–5: toes 2–5, M1: first metatarsal, M2: second metatarsal, M3: third metatarsal, M4: fourth metatarsal, M5: fifth metatarsal, MF: midfoot, MH: medial heel, and LH: lateral heel.

**Table 2:** Regional intersession ICCs and CVs for plantar loading measures in the WOT and WT protocols.

Variable	Zone	WOT		WT	
		ICCs	CVs	ICCs	CVs
PP	T1	0.882	12.6	0.902	15.6
	T2–5	0.841	16.8	0.789	19.2
	M1	0.805	13.1	0.822	12.4
	M2	0.874	8.9	0.962	6.7
	M3	0.919	9.8	0.903	13.2
	M4	0.824	10.6	0.757	7.2
	M5	0.774	10.8	0.861	3.8
	MF	0.762	16.2	0.770	17.3
	MH	0.857	8.2	0.793	9.5
	LH	0.889	7.8	0.814	8.7

CT	T1	0.914	9.0	0.842	12.0
	T2–5	0.870	9.1	0.667	13.1
	M1	0.879	8.2	0.813	7.8
	M2	0.886	1.8	0.827	6.3
	M3	0.818	1.3	0.715	1.5
	M4	0.839	1.3	0.893	1.7
	M5	0.806	2.0	0.828	2.0
	MF	0.919	4.1	0.882	5.1
	MH	0.868	3.8	0.830	4.9
	LH	0.870	3.9	0.854	5.0

CA	T1	0.923	3.7	0.907	4.9
	T2–5	0.816	8.4	0.794	13.1
	M1	0.907	5.9	0.916	5.7
	M2	0.872	4.0	0.764	5.5
	M3	0.804	4.7	0.826	4.3
	M4	0.882	3.9	0.670	5.6
	M5	0.813	5.1	0.728	5.5
	MF	0.965	3.8	0.983	3.0
	MH	0.960	2.4	0.798	3.2
	LH	0.945	2.7	0.898	2.5

PTI	T1	0.753	19.0	0.826	22.5
	T2–5	0.781	23.0	0.778	25.4
	M1	0.799	14.7	0.822	15.9
	M2	0.858	9.2	0.788	14.0
	M3	0.819	10.3	0.802	12.2
	M4	0.803	12.5	0.814	14.6
	M5	0.789	12.4	0.833	15.5
	MF	0.824	18.3	0.773	20.1
	MH	0.800	13.1	0.851	14.7
	LH	0.903	9.3	0.787	10.7

ICCs: intraclass correlation coefficients, CVs: coefficient of variations, WOT: without top-layer, WT: with top-layer, PP: peak pressure, CT: contact time, CA: contact area, PTI: pressure-time integral, T1: hallux, T2–5: toes 2–5, M1: first metatarsal, M2: second metatarsal, M3: third metatarsal, M4: fourth metatarsal, M5: fifth metatarsal, MF: midfoot, MH: medial heel, and LH: lateral heel.

**Table 3:** Comparison of the PP, CT, CA, and PTI in the 10 masked zones between sessions in WOT protocol.

Zone	PP (kPa)			CT (stance time%)			CA (cm^2^)			PTI (kPa s)		
	Session 1	Session 2	*P*	Session 1	Session 2	*P*	Session 1	Session 2	*P*	Session 1	Session 2	*P*
T1	165.5 ± 51.7	157.7 ± 46.1	0.201	57.6 ± 15.6	57.8 ± 15.4	0.894	15.5 ± 2.3	15.5 ± 2.5	0.829	46.1 ± 18.2	34.7 ± 14.2	0.146
T2–5	50.2 ± 25.1	44.0 ± 19.5	0.226	41.8 ± 11.8	41.8 ± 12.2	0.985	16.2 ± 3.0	15.8 ± 3.5	0.387	9.2 ± 4.5	7.8 ± 3.4	0.299
M1	166.4 ± 42.2	190.2 ± 34.5	0.184	68.2 ± 12.2	68.7 ± 13.4	0.783	13.4 ± 2.9	13.2 ± 2.4	0.414	42.9 ± 15.7	48.5 ± 16.3	0.645
M2	380.9 ± 91.8	354.0 ± 84.2	0.068	79.9 ± 4.6	79.9 ± 4.1	0.951	11.5 ± 1.7	11.7 ± 1.3	0.345	90.8 ± 26.4	86.0 ± 23.6	0.630
M3	349.3 ± 105.8	339.9 ± 97.0	0.154	82.9 ± 3.9	82.8 ± 3.6	0.854	13.8 ± 0.9	11.2 ± 1.4	0.254	87.7 ± 30.6	82.7 ± 25.6	0.482
M4	245.1 ± 61.7	224.0 ± 50.9	0.091	82.2 ± 3.5	82.3 ± 4.3	0.880	9.7 ± 0.9	9.9 ± 1.5	0.082	55.2 ± 21.9	54.2 ± 18.7	0.328
M5	119.0 ± 33.8	113.8 ± 28.6	0.465	77.9 ± 4.8	77.7 ± 5.2	0.804	12.9 ± 1.7	12.8 ± 2.2	0.849	33.0 ± 15.5	30.8 ± 13.6	0.617
MF	64.6 ± 30.2	65.8 ± 24.6	0.718	62.9 ± 10.0	63.0 ± 8.4	0.176	38.4 ± 7.6	38.4 ± 7.8	0.959	16.7 ± 8.1	14.9 ± 6.3	0.469
MH	253.0 ± 54.1	258.4 ± 46.2	0.229	59.2 ± 5.5	58.2 ± 7.4	0.180	21.9 ± 2.2	21.8 ± 2.7	0.490	57.5 ± 18.1	48.5 ± 14.9	0.303
LH	220.5 ± 48.2	219.5 ± 43.2	0.838	57.7 ± 6.0	58.1 ± 7.3	0.245	19.3 ± 2.1	19.3 ± 2.2	0.885	46.7 ± 15.9	45.5 ± 12.7	0.581

Values are expressed as means ± standard deviation; WOT: without top-layer, PP: peak pressure, CT: contact time, CA: contact area, PTI: pressure-time integral, T1: hallux, T2–5: toes 2–5, M1: first metatarsal, M2: second metatarsal, M3: third metatarsal, M4: fourth metatarsal, M5: fifth metatarsal, MF: midfoot, MH: medial heel, and LH: lateral heel.

**Table 4:** Comparison of the PP, CT, CA, and PTI in the 10 masked zones between sessions in WT protocol.

Zone	PP (kPa)			CT (stance time%)			CA (cm^2^)			PTI (kPa s)		
	Session 1	Session 2	*P*	Session 1	Session 2	*P*	Session 1	Session 2	*P*	Session 1	Session 2	*P*
T1	136.3 ± 44.7	141.1 ± 48.1	0.772	54.4 ± 16.4	52.4 ± 16.7	0.396	15.5 ± 3.2	16.0 ± 3.7	0.113	38.0 ± 14.9	30.8 ± 11.3	0.105
T2–5	41.7 ± 19.1	44.7 ± 22.5	0.187	34.9 ± 11.7	35.7 ± 14.1	0.575	13.6 ± 5.2	12.8 ± 4.3	0.149	5.2 ± 3.2	6.0 ± 3.8	0.264
M1	166.4 ± 35.9	158.3 ± 38.1	0.314	65.5 ± 14.3	67.0 ± 15.2	0.417	12.3 ± 2.2	12.4 ± 2.8	0.647	46.8 ± 16.0	38.6 ± 13.2	0.221
M2	332.2 ± 78.7	330.8 ± 76.5	0.781	78.2 ± 5.9	78.9 ± 7.3	0.377	11.0 ± 2.3	11.3 ± 2.6	0.368	88.2 ± 25.4	79.8 ± 22.6	0.716
M3	310.5 ± 87.0	326.4 ± 93.4	0.201	82.5 ± 4.7	82.2 ± 4.1	0.557	11.9 ± 1.3	11.9 ± 1.5	0.752	87.7 ± 29.1	76.5 ± 26.3	0.350
M4	218.6 ± 59.2	204.8 ± 54.8	0.171	82.1 ± 7.1	81.7 ± 5.9	0.395	9.3 ± 1.3	9.2 ± 1.5	0.618	45.9 ± 19.8	42.5 ± 18.2	0.479
M5	113.8 ± 41.8	114.2 ± 39.6	0.848	77.4 ± 5.5	77.7 ± 4.3	0.589	12.8 ± 2.0	12.8 ± 2.1	0.860	31.8 ± 14.3	25.8 ± 12.7	0.770
MF	60.4 ± 30.9	57.4 ± 28.3	0.324	62.6 ± 10.1	62.1 ± 11.5	0.494	38.4 ± 8.2	38.5 ± 9.4	0.712	13.3 ± 7.6	10.8 ± 6.2	0.131
MH	227.5 ± 43.0	219.1 ± 41.2	0.168	58.9 ± 6.0	58.0 ± 7.2	0.469	22.5 ± 2.6	22.8 ± 2.9	0.399	44.3 ± 11.5	49.7 ± 12.7	0.764
LH	202.2 ± 43.3	190.4 ± 34.5	0.073	57.5 ± 5.6	56.8 ± 7.1	0.321	19.9 ± 1.9	19.9 ± 1.9	0.948	41.8 ± 13.5	40.6 ± 11.3	0.812

Values are expressed as means ± standard deviation; WT: with top-layer, PP: peak pressure, CT: contact time, CA: contact area, PTI: pressure-time integral, T1: hallux, T2–5: toes 2–5, M1: first metatarsal, M2: second metatarsal, M3: third metatarsal, M4: fourth metatarsal, M5: fifth metatarsal, MF: midfoot, MH: medial heel, and LH: lateral heel.

**Table 5:** Comparison of the PP (kPa) in the 10 masked zones between the WOT and WT protocols.

Zone	WOT	WT	*P*	AD	PD (%)
T1	161.6 ± 48.9	138.7 ± 46.4	0.002^*∗*^	22.9	14.2
T2–5	47.1 ± 22.3	43.2 ± 20.8	0.266	3.9	8.3
M1	178.3 ± 38.3	162.3 ± 37.0	0.155	16.0	9.0
M2	367.5 ± 87.9	331.5 ± 77.5	<0.001^*∗*^	36.0	9.8
M3	344.6 ± 101.4	318.4 ± 90.2	<0.001^*∗*^	26.2	7.6
M4	234.6 ± 56.3	211.7 ± 57.0	0.005^*∗*^	22.9	9.8
M5	116.4 ± 31.2	114.0 ± 40.7	0.868	2.4	2.1
MF	65.3 ± 27.3	58.9 ± 29.5	0.006^*∗*^	6.4	9.8
MH	255.7 ± 50.1	223.3 ± 42.1	<0.001^*∗*^	32.4	12.7
LH	220.0 ± 45.7	196.3 ± 38.9	<0.001^*∗*^	23.7	10.8

^*∗*^ *P* < 0.05; values are expressed as means ± standard deviation; PP: peak pressure, WOT: without top-layer, WT: with top-layer, AD: absolute differences, PD: percentage differences, T1: hallux, T2–5: toes 2–5, M1: first metatarsal, M2: second metatarsal, M3: third metatarsal, M4: fourth metatarsal, M5: fifth metatarsal, MF: midfoot, MH: medial heel, and LH: lateral heel.

**Table 6:** Comparison of the CT (stance time%) in the 10 zones between the WOT and WT protocols.

Zone	WOT	WT	*P*	AD	PD (%)
T1	57.7 ± 15.5	53.4 ± 16.5	0.001^*∗*^	4.3	7.5
T2–5	41.8 ± 12.0	35.3 ± 12.9	<0.001^*∗*^	6.5	15.6
M1	68.5 ± 12.8	66.3 ± 14.7	0.027^*∗*^	2.2	3.2
M2	79.9 ± 4.3	78.6 ± 6.6	0.380	1.3	1.6
M3	82.8 ± 3.7	82.4 ± 4.4	0.293	0.4	0.5
M4	82.2 ± 3.9	81.9 ± 6.5	0.590	0.3	0.4
M5	77.8 ± 5.0	77.6 ± 4.9	0.595	0.2	0.3
MF	62.9 ± 9.2	62.3 ± 10.8	0.842	0.6	1.0
MH	58.7 ± 6.4	58.5 ± 6.5	0.570	0.2	0.3
LH	57.9 ± 6.6	57.2 ± 6.3	0.810	0.7	1.2

^*∗*^ *P* < 0.05; values are expressed as means ± standard deviation; CT: contact time, WOT: without top-layer, WT: with top-layer, AD: absolute differences, PD: percentage differences, T1: hallux, T2–5: toes 2–5, M1: first metatarsal, M2: second metatarsal, M3: third metatarsal, M4: fourth metatarsal, M5: fifth metatarsal, MF: midfoot, MH: medial heel, and LH: lateral heel.

**Table 7:** Comparison of the CA (cm^2^) in the 10 masked zones between the WOT and WT protocols.

Zone	WOT	WT	*P*	AD	PD (%)
T1	15.5 ± 2.4	15.8 ± 3.4	0.343	−0.3	−1.9
T2–5	16.0 ± 3.2	13.2 ± 4.7	<0.001^*∗*^	2.8	17.5
M1	13.3 ± 2.6	12.4 ± 2.5	<0.001^*∗*^	0.9	6.8
M2	11.6 ± 1.5	11.1 ± 2.4	0.022^*∗*^	0.5	4.3
M3	12.5 ± 1.1	11.9 ± 1.4	0.001^*∗*^	0.6	4.8
M4	9.8 ± 1.2	9.2 ± 1.3	<0.001^*∗*^	0.6	6.1
M5	12.8 ± 1.9	12.8 ± 2.0	0.611	0.0	0.0
MF	38.4 ± 7.7	38.4 ± 8.8	0.896	0.0	0.0
MH	21.8 ± 2.4	22.6 ± 2.7	0.087	−0.8	−3.7
LH	19.3 ± 2.1	19.9 ± 1.9	0.452	−0.6	−3.1

^*∗*^ *P* < 0.05; values are expressed as means ± standard deviation; CA: contact area, WOT: without top-layer, WT: with top-layer, AD: absolute differences, PD: percentage differences, T1: hallux, T2–5: toes 2–5, M1: first metatarsal, M2: second metatarsal, M3: third metatarsal, M4: fourth metatarsal, M5: fifth metatarsal, MF: midfoot, MH: medial heel, and LH: lateral heel.

**Table 8:** Comparison of the PTI (kPa s) in the 10 masked zones between the WOT and WT protocols.

Zone	WOT	WT	*P*	AD	PD (%)
T1	40.4 ± 16.2	34.4 ± 13.1	0.028^*∗*^	6.0	14.9
T2–5	8.5 ± 3.9	5.6 ± 3.5	0.032^*∗*^	2.9	34.1
M1	45.7 ± 15.9	42.7 ± 14.6	0.599	3.0	6.6
M2	88.3 ± 25.0	84.0 ± 24.1	0.792	4.3	4.9
M3	85.2 ± 28.1	82.1 ± 27.7	0.088	3.1	3.6
M4	54.7 ± 20.3	44.2 ± 18.9	<0.001^*∗*^	10.5	19.2
M5	31.9 ± 14.5	28.8 ± 13.5	0.407	3.1	9.7
MF	15.8 ± 7.1	12.0 ± 6.9	0.747	3.8	24.1
MH	53.0 ± 16.5	47.0 ± 12.1	<0.001^*∗*^	6.0	11.3
LH	46.1 ± 14.2	41.2 ± 12.4	0.003^*∗*^	4.9	10.6

^*∗*^ *P* < 0.05; values are expressed as means ± standard deviation; PTI: pressure-time integral, WOT: without top-layer, WT: with top-layer, AD: absolute differences, PD: percentage differences, T1: hallux, T2–5: toes 2–5, M1: first metatarsal, M2: second metatarsal, M3: third metatarsal, M4: fourth metatarsal, M5: fifth metatarsal, MF: midfoot, MH: medial heel, and LH: lateral heel.

## References

[1] Chang C.-F., Wang T.-M., Wang J.-H., Huang S.-C., Lu T.-W. (2012). Residual gait deviations in adolescents treated during infancy for unilateral developmental dysplasia of the hip using Pemberton's osteotomy. *Gait and Posture*.

[2] Hähni M., Hirschmüller A., Baur H. (2016). The effect of foot orthoses with forefoot cushioning or metatarsal pad on forefoot peak plantar pressure in running. *Journal of Foot and Ankle Research*.

[3] Franklyn-Miller A., Bilzon J., Wilson C., McCrory P. (2014). Can RSScan footscan® D3D™ software predict injury in a military population following plantar pressure assessment? A prospective cohort study. *Foot*.

[4] Deschamps K., Matricali G. A., Desmet D. (2016). Efficacy measures associated to a plantar pressure based classification system in diabetic foot medicine. *Gait and Posture*.

[5] Salazar-Torres J. J., McDowell B. C., Humphreys L. D., Duffy C. M. (2014). Plantar pressures in children with congenital talipes equino varus-A comparison between surgical management and the Ponseti technique. *Gait and Posture*.

[6] Van Der Leeden M., Dekker J. H. M., Siemonsma P. C., Lek-Westerhof S. S., Steultjens M. P. M. (2004). Reproducibility of plantar pressure measurements in patients with chronic arthritis: A comparison of one-step, two-step, and three-step protocols and an estimate of the number of measurements required. *Foot and Ankle International*.

[7] Zammit G. V., Menz H. B., Munteanu S. E. (2010). Reliability of the TekScan MatScan® system for the measurement of plantar forces and pressures during barefoot level walking in healthy adults. *Journal of Foot and Ankle Research*.

[8] Maetzler M., Bochdansky T., Abboud R. J. (2010). Normal pressure values and repeatability of the Emed® ST2 system. *Gait and Posture*.

[9] Ramanathan A. K., Kiran P., Arnold G. P., Wang W., Abboud R. J. (2010). Repeatability of the Pedar-X® in-shoe pressure measuring system. *Foot and Ankle Surgery*.

[10] Murphy D. F., Beynnon B. D., Michelson J. D., Vacek P. M. (2005). Efficacy of plantar loading parameters during gait in terms of reliability, variability, effect of gender and relationship between contact area and plantar pressure. *Foot and Ankle International*.

[11] Putti A. B., Arnold G. P., Cochrane L., Abboud R. J. (2007). The Pedar® in-shoe system: Repeatability and normal pressure values. *Gait and Posture*.

[12] Castro M. P. D., Meucci M., Soares D. P. (2014). Accuracy and repeatability of the gait analysis by the walkinsense system. *BioMed Research International*.

[13] Putti A. B., Arnold G. P., Cochrane L. A., Abboud R. J. (2008). Normal pressure values and repeatability of the Emed ST4 system. *Gait and Posture*.

[14] Gurney J. K., Kersting U. G., Rosenbaum D. (2008). Between-day reliability of repeated plantar pressure distribution measurements in a normal population. *Gait and Posture*.

[15] Martínez-Nova A., Cuevas-García J. C., Pascual-Huerta J., Sánchez-Rodríguez R. (2007). BioFoot® in-shoe system: Normal values and assessment of the reliability and repeatability. *Foot*.

[16] Xu C., Wen X., Huang L. (2017). Normal foot loading parameters and repeatability of the Footscan® platform system. *Journal of Foot and Ankle Research*.

[17] Rietdyk S., Drifmeyer J. E. (2009). The rough-terrain problem: Accurate foot targeting as a function of visual information regarding target location. *Journal of Motor Behavior*.

[18] Patla A. E., Adkin A., Martin C., Holden R., Prentice S. (1996). Characteristics of voluntary visual sampling of the environment for safe locomotion over different terrains. *Experimental Brain Research*.

[19] Verniba D., Vergara M. E., Gage W. H. (2015). Force plate targeting has no effect on spatiotemporal gait measures and their variability in young and healthy population. *Gait and Posture*.

[20] Grabiner M. D., Feuerbach J. W., Lundin T. M., Davis B. L. (1995). Visual guidance to force plates does not influence ground reaction force variability. *Journal of Biomechanics*.

[21] Wearing S. C., Urry S. R., Smeathers J. E. (2000). The effect of visual targeting on ground reaction force and temporospatial parameters of gait. *Clinical Biomechanics*.

[22] Xu C., Yan Y.-B., Zhao X. (2015). Pedobarographic Analysis Following Pemberton's Pericapsular Osteotomy for Unilateral Developmental Dysplasia of the Hip: An Observational Study. *Medicine (United States)*.

[23] Huang H., Qiu J., Liu T. (2017). Similarity of center of pressure progression during walking and jogging of Anterior Cruciate Ligament deficient patients. *PLoS ONE*.

[24] Vallejo R. B. D. B., Iglesias M. E. L., Zeni J., Thomas S. (2013). Reliability and repeatability of the portable EPS-platform digital pressure-plate system. *Journal of the American Podiatric Medical Association*.

[25] Menz H. B. (2005). Analysis of Paired Data in Physical Therapy Research: Time to Stop Double-Dipping?. *Journal of Orthopaedic & Sports Physical Therapy*.

[26] Bryant A. R., Tinley P., Singer K. P. (2000). Normal values of plantar pressure measurements determined using the EMED-SF system. *Journal of the American Podiatric Medical Association*.

[27] Hafer J. F., Lenhoff M. W., Song J., Jordan J. M., Hannan M. T., Hillstrom H. J. (2013). Reliability of plantar pressure platforms. *Gait and Posture*.

[28] Atkinson G., Nevill A. M. (1998). Statistical methods for assessing measurement error (reliability) in variables relevant to sports medicine. *Sports Medicine*.

[29] Peters E. J. G., Urukalo A., Fleischli J. G., Lavery L. A. (2002). Reproducibility of gait analysis variables: One-step versus three-step method of data acquisition. *Journal of Foot and Ankle Surgery*.

[30] Cornwall M., McPoil T. (1997). The effect of foot orthotics on the initiation of plantar surface loading. *Clinical Biomechanics*.

[31] Nagel A., Fernholz F., Kibele C., Rosenbaum D. (2008). Long distance running increases plantar pressures beneath the metatarsal heads. A barefoot walking investigation of 200 marathon runners. *Gait and Posture*.

[32] Menz H. B., Zammit G. V., Munteanu S. E. (2007). Plantar pressures are higher under callused regions of the foot in older people. *Clinical and Experimental Dermatology*.

